# Ovarian Transcriptome Profile from Egg-Laying Period to Incubation Period of Changshun Green-Shell Laying Hens

**DOI:** 10.3390/genes16040394

**Published:** 2025-03-29

**Authors:** Zhi Chen, Di Wen

**Affiliations:** College of Biological Science and Agriculture, Qiannan Normal University for Nationalities, Duyun 558000, China; wendyy2007@163.com

**Keywords:** transcriptome analysis, ovary, laying period, incubation period, Changshun green-shell laying hen

## Abstract

**Background/Objectives:** The Changshun green-shell laying hen with a strong broodiness is a Chinese indigenous chicken breed. Little is known about the mechanisms responsible for the ovary development of Changshun green-shell laying hens from the egg-laying period (LP) to the incubation period (BP). **Methods:** A total of six hens were selected from LP (*n* = three) and BP (*n* = three) at 28 weeks old. The RNA sequencing (RNA-seq) of ovaries from hens in LP and BP groups was performed to identify candidate genes and pathways associated with broodiness. **Results:** We identified 1650 differently expressed genes (DEGs), including 429 up-regulated and 1221 down-regulated DEGs, in chicken ovaries between LP and BP groups. Gene ontology term (GO) and Kyoto Encyclopedia of Genes and Genomes (KEGG) analysis revealed that these DEGs were mainly involved in the pathways related to follicle development in chicken ovaries, including focal adhesion, the MAPK signaling pathway, and the FoxO signaling pathway, and vascular smooth muscle contraction, ECM–receptor interaction, and the GnRH signaling pathway were down-regulated in incubating ovaries. Eight candidate genes (*EGFR*, *VEGFRKDRL*, *FLT1*, *KDR*, *PDGFRA*, *TEK*, *KIT* and *FGFR3*) related to angiogenesis, folliculogenesis, steroidogenesis and oogenesis in ovaries were suggested to play important roles in the ovarian development of Changshun hens during the transition from LP to BP. **Conclusions:** This study identified a range of genes and several pathways that may be involved in regulating the broodiness of Changshun green-shell laying hens. These data are helpful to further enrich our understanding of the mechanism of incubation behaviour in chickens.

## 1. Introduction

Incubation behaviour is a common maternal behaviour in domestic chickens. It causes the cessation of egg laying, which results in economic losses for the poultry industry. Therefore, the mechanism that controls broodiness has interested poultry scientists for a century. Although the regulatory regulation mechanism of avian broodiness remains unclear, it is generally believed that broodiness is mainly controlled by the central nervous system and is closely associated with multiple hormones secreted by the hypothalamic-pituitary-gonadal axis (HPGA), especially pituitaric prolactin (PRL) [1,2,3]. Briefly, incubation behaviour is triggered by various external stimuli, such as ambient temperature, photoperiod and the presence of eggs, that act on the central nervous system, resulting in a surge in plasma PRL levels. Pituitaric PRL has a specific role in regulating avian incubation behaviour. A characteristic increase of PRL in plasma is critical for the onset and maintain of chicken broodiness [4,5,6,7]. Subsequently, PRL levels in chicken plasma rapidly decline after hatching. In addition to PRL as a critical mediator of broodiness, luteinizing hormone, hypothalamic gonadotropin-releasing hormone (GnRH), neuropeptide vasoactive intestinal polypeptide, neurotransmitter dopamine, 5-hydroxytryptophan and dynorphin are also reported to be involved in chicken broodiness [6,8,9,10,11,12,13,14].

Ovaries play an important role in determining the activities of the hypothalamic-pituitary unit. Therefore, understanding the alterations in specific genes and signaling pathways between the egg-laying period (LP) and the incubation period (BP) is essential to unravel the underlying mechanisms of broodiness in poultry. The advent of high-throughput sequencing technologies, including RNA sequencing (RNA-Seq), has introduced novel approaches, such as genomic analyses, that offer a robust means of elucidating the mechanisms that influence avian broodiness. Although genomic analyses based on high-throughput sequencing technologies show that an increasing number of candidate genes and signaling pathways in ovaries are reported to be involved in the broodiness of poultry, there have been no specific genes and pathways elucidated which have marked effects on incubation behaviour. Different breeds or strains of poultry exhibit variation in ovarian genes expression and signaling pathways related to incubation behaviour, indicating that there is a genetic basis to incubation behaviour in poultry [15,16,17,18,19,20,21,22]. In incubating Muscovy ducks (*Cairina moschata*), a total of 334 differential expression genes (DEGs) and 36 differentially abundant long noncoding RNAs (lncRNAs) transcripts were identified in ovaries, and focal adhesion, the FOXO signaling pathway, Wnt signaling, oocyte meiosis and the cytokine–cytokine receptor interaction pathways in ovaries might involve in the transition from egg-laying to incubation [15]. However, differentially expressed mRNAs and the target genes of differentially expressed lncRNAs in the ovaries of Taihe Black-Bone Silky (*Gallus gallus Domesticus Brisson*) are mainly associated with neuroactive ligand–receptor interaction, CCR6 chemokine receptor binding, G-protein coupled receptor binding, cytokine–cytokine receptor interaction, and ECM–receptor interaction [16]. iTRAQ-based quantitative proteomic analysis revealed that APOV1, GAL, SAA, GNB5, VLDLR and CDK1 might be the key molecules involved in the regulation of incubation behaviours in Muscovy ducks [17]. In Zhedong white geese (*Anser cygnoides*), 572 DEGs were identified in ovaries between LP and BP geese, but FSHβ, PRL and PRLR were not observed to be expressed differentially [18]. Further analysis indicated that these DEGs are mainly associated with reproduction regulation, such as steroid hormone biosynthesis, the GnRH signaling pathway, the calcium signaling pathway, the Wnt signaling pathway and oocyte meiosis [18]. Meanwhile, transcriptomes of different types of follicles, including small white, large white and small yellow follicles, indicated that most of the DEGs are involved in hormone response, autophagy, follicular development and oxidation [19,20]. Additionally, FOS, HSP90AA and CDK1 in ovaries are found to consolidate and transduce signals that regulate the HPGA during broodiness in Tianfu meat geese [21]. Focal adhesion, ECM–receptor interaction and N-Glycan biosynthesis were enriched significantly in the ovaries of Xupu geese during the pre-laying period, laying period and incubating period [22]. These results further indicated the complexity of the javascript:void(0);reproductive behaviour of different avian breeds and strains. Further efforts, including the study of the transcriptome profile of the ovaries from different avian breeds and strains, seem to be essential for elucidating the mechanisms of incubation behaviour in poultry.

The Changshun green-shell laying hen is a Chinese indigenous chicken breed with strong broodiness. Due to its strong tendency for broodiness, the Changshun hen has recently become an important model for studying avian incubation behaviour [23]. Nevertheless, changes in the mRNA expression profile in the ovaries of Changshun green-shell laying hens from the LP to BP have not been reported. Therefore, this study aimed to comprehensively analyze and compare the transcriptome profiles of the ovaries between the LP and BP groups using RNA-seq. Candidate genes and signal pathways related to the broodiness of Changshun hens were identified through the Kyoto Encyclopedia of Genes and Genomes (KEGG) and gene ontology term (GO) enrichment analyses. It is hypothesized that several unique genes and pathways in ovaries which are different from other poultry breeds might be involved in the broodiness of Changshun hens. The results based on Changshun green-shell laying hens will not only provide new insights on the ovarian regulation of incubation behaviour, but also further confirm the complexity of the javascript:void(0);reproductive behaviour of different avian breeds.

## 2. Materials and Methods

### 2.1. Ethics Statement

Animal experimental protocols employed in this study were performed in accordance with the guidelines formulated by the Ministry of Science and Technology of the People’s Republic of China. All animal experiments of this study were approved by the Animal Ethics Committee of the College of Biological Science and Agriculture, Qiannan Normal University for Nationalities (AEC No. QNUN2021014).

### 2.2. Sample Collection

A total of 100 4-week-old chicks were purchased from Changshun Sanyuan Agricultural Development Co., Ltd., Changshun, China and were then raised in the poultry breeding farm of Qiannan Normal University for Nationalities. The animals were reared on the floor with litter and artificial nests and exposed to natural temperature conditions (18~24 °C). The relative humidity was 55~75%. Lighting was provided 13 h light and 11 h darkness. The animals were allowed ad libitum access to water and fed with diet three times daily (7:30, 13:30 and 18:30). The ingredients and nutrient composition of the diets are shown in Table S1. The houses were routinely disinfected according to a schedule to maintain appropriate standards of cleanliness. The hens with high fertility were allocated as the LP group. The individuals which sat in the nest while exhibiting characteristic clucking, defensive or aggressive behaviour for at least one week were deemed to be BP hens. Finally, a total of 6 Changshun hens, including 3 incubating and 3 egg-laying hens, were collected at 28 weeks of age according to their behaviour, respectively. Changshun hens were euthanized with exsanguination under sodium pentobarbital anesthesia (60 mg/kg, 5 min), which was performed by competent personnel who experienced and correctly applied the technique to minimize any pain or distress to the hens. Stroma with cortical follicles < 1 mm in diameter were collected swiftly. The morphologic characteristics of the ovaries were used to further evaluate the physiological stage of Changshun hens. The laying hens were mainly distinguished by the presence of hierarchical follicles on the ovaries. On the contrary, the ovaries of incubating Changshun hens lacked hierarchical follicles. All the ovarian samples were frozen in liquid nitrogen, and then transferred to −80 °C until analysis.

### 2.3. RNA Isolation and Sequencing

Total RNA was extracted from ovaries using the Trizol reagent (Life technologies, Carlsbad, CA, USA). The concentration and quality of the total RNA were determined using the NanoDrop 2000 (Thermo Fisher Scientific, Wilmington, DE, USA) and electrophoresis. The OD260/OD280 ratio of the RNA fell within the range of 1.8–2.1, indicating high RNA purity and the absence of significant impurities, making the samples suitable for further experiments. The integrity of ovarian samples was assessed using the RNA Nano 6000 Assay Kit of the Agilent Bioanalyzer 2100 system (Agilent Technologies, Santa Clara, CA, USA). An RNA integrity number value of >8 for total RNA is desirable for RNA-Seq library preparation. The resulting libraries in this study were then sequenced on an Illumina NovaSeq6000 platform (Illumina, San Diego, CA, USA) using 150 bp paired-end read mode. RNA-Seq was performed using six biological replicates, with no technical replicates for each biological replicate.

### 2.4. Principal Component Analysis and Differential Expression Analysis

The raw reads were processed with a bioinformatics pipelinetool, BMKCloud (www.biocloud.net) online platform. Firstly, raw sequences were transformed into clean reads by discarding low-quality sequences and adaptor sequences. Subsequently, Hisat2 (v2.2.1, -dta -p 6 -max-intronlen 5,000,000) was applied to construct the reference genome index and align the clean data to the reference genome (Gallus_gallus.GRCg6a_release106.genome.fa) [24]. All transcripts from Hisat2 alignment results were detected using the method of the StringTie Reference Annotation Based Transcript (v2.2.1, -merge -F 0.1 -T 0.1) [25]. The gene function was annotated with the National Center for Biotechnology Information for non-redundant proteins (ftp://ftp.ncbi.nih.gov/blast/db/, accessed on 6 June 2024), KEGG (http://www.genome.jp/kegg/, accessed on 6 June 2024), GO (http://www.geneontology.org/, accessed on 6 June 2024) and Swiss-Prot (http://www.uniprot.org/, accessed on 6 June 2024) databases. The similarity between the LP and BP groups was assessed using principal component analysis (PCA). DEGs between LP and BP groups were identified using the DESeq2 package (v1.30.1) [26]. DESeq2 provides statistical routines for determining differential expression in digital gene expression data using a model based on the negative binomial distribution. |log_2_FC| ≥ 1.5 and False Discovery Rate (FDR)-corrected *p* < 0.01 were set as the criteria for DEGs.

### 2.5. Enrichment Analysis and Protein–Protein Interaction

GO was used to enrich molecular functions, cellular components, and biological processes, and KEGG was used to enrich key pathways of DEGs. The clusterProfiler package (v3.16.1) was used to test the statistical enrichment of DEGs in GO terms and KEGG pathways [27,28]. Cytoscape (v3.5.1) was used to perform the protein–protein interaction (PPI) analysis [29]. Circle and rectangle nodes represent a protein and a KEGG pathway, respectively. The solid and dashed lines represent an interaction between two proteins and the edge of KEGG pathway in PPI network, respectively.

### 2.6. Gene Expression Analysis Using Quantitative Real-Time PCR

Four DEGs, including ANGPT2, TEK, EGFR and PTEN, were selected randomly to further validate the results of RNA-seq by quantitative real-time PCR (qRT-PCR). Table S2 listed the primers that were designed with Primer 5. The primers were validated for specificity and efficiency. The protocol of qRT-PCR was initiated at 95 °C for 10 min, followed by 40 cycles of the amplification program, with denaturation at 95 °C for 15 s and annealing/extension at 60 °C for 60 s. Melt curves were generated at the end of the last amplification cycle to confirm the specificity of the amplification reaction. All reactions were run in triplicate. The relative mRNA expression levels were estimated using the 2^−ΔΔCt^ method and normalized using *β-actin* [30]. Data was analyzed using the Student’s *t*-test after testing for the homogeneity of variance with Levene’s test. All data are presented as the mean ±  SD, and statistical significance was shown as * *p* < 0.05.

## 3. Results

### 3.1. RNA Sequencing Quality Assessment and Transcriptome Alignment

Total RNA was extracted from the ovaries of Changshun green-shell laying hens at the LP and BP. Six cDNA sequencing libraries (three egg-laying period samples and three incubation period samples) were sequenced on the Illumina NovaSeq6000 platform. As shown in Table 1, more than 19.2 × 10^6^ clean reads per ovarian sample were generated after filtering. The base percentage of the Q20 and Q30 was above 98.34% and 94.87%, respectively, and the GC content of six ovarian samples ranged from 47.89% to 51.22%. More than 93.16% of clean reads were perfectly mapped to the reference genome of chicken to generate a read count value. The percentage of uniquely and multiple mapped reads in clean reads ranged from 90.60% to 93.84% and 1.81% to 2.43%, respectively. The results indicated that the transcriptome data were suitable for subsequent analysis.

### 3.2. Differential Expressed Analysis

**Figure 1:**
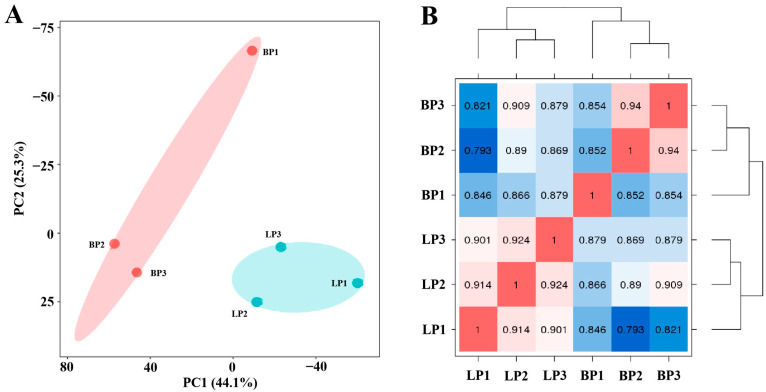
Features of sequencing data. (**A**) PCA score plot of ovarian transcriptomes. Red and green nodes represent individuals from the BP and LP, respectively. (**B**) Pearson correlation analysis of the LP and BP groups. LP1, LP2 and LP3 are ovarian samples from egg-laying hens, and BP1, BP2 and BP3 are ovarian samples from incubation hens. Colour indicates the Pearson correlation value.

**Figure 2:**
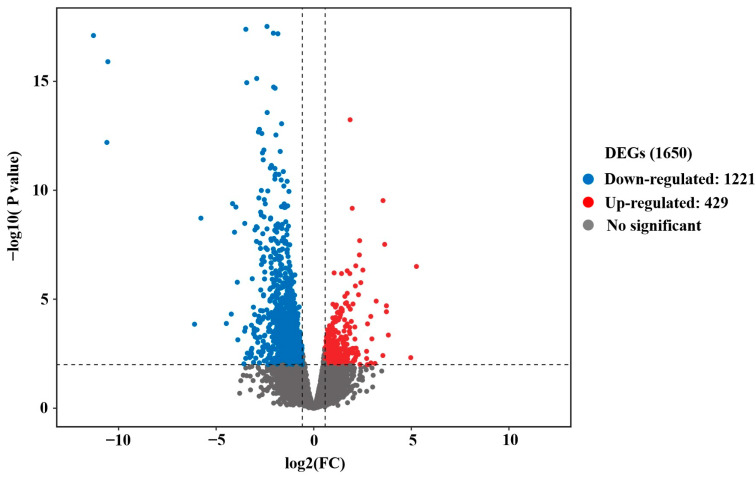
Volcano map of all expressed genes. The horizontal and longitudinal coordinates represent the fold changes of genes and the statistical significance of the changes in gene expression, respectively. Blue and red plots represent significantly up- and down-regulated genes (|log_2_FC| ≥ 1.5, FDR-corrected *p* value < 0.01), respectively. Black plots are genes without significant difference.

Firstly, the ovarian samples were analyzed by performing PCA. As shown in Figure 1A, six ovarian samples were divided into two parts in PCA, indicating an obvious difference between the LP and BP groups. In accordance with the results of PCA, good sample repeatability was observed in the ovarian transcriptome (Figure 1B), indicating the reliable and reasonable analysis of DEGs in the following study. The cutoff criteria were |log_2_FC| ≥ 1.5, FDR-corrected *p* value < 0.01. Further analysis using the DESeq2 package revealed that a total of 1650 DEGs were identified in chicken ovaries, including 429 up-regulated and 1221 down-regulated genes (Figure 2 and Table S3). The hierarchical clustering analysis of DEGs showed that ovarian samples from the same group were clustered together, and a heatmap was then used to visualize the expression patterns of genes in ovaries between the LP and BP groups (Figure S1).

### 3.3. KEGG Pathway and GO Enrichment Analysis

**Figure 3:**
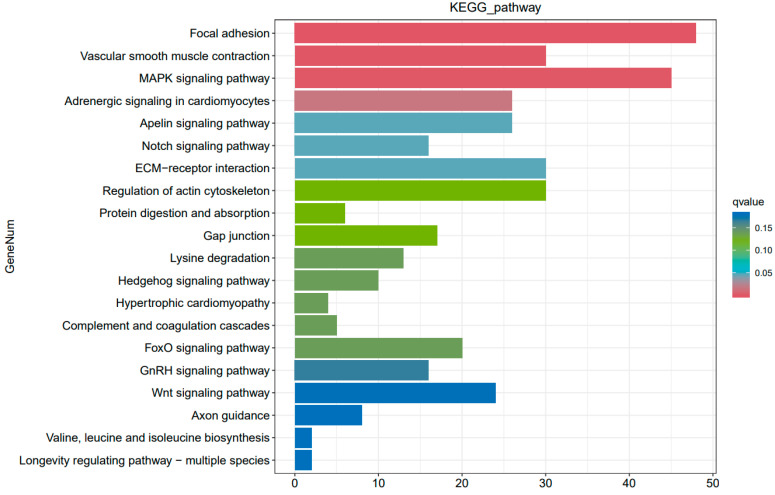
Top 20 KEGG pathways enriched by the ovarian DEGs. The longitudinal and horizontal coordinates represent the name of the pathway and the number of pathway corresponding DEGs, respectively. The color corresponds to the q value of each pathway.

**Figure 4:**
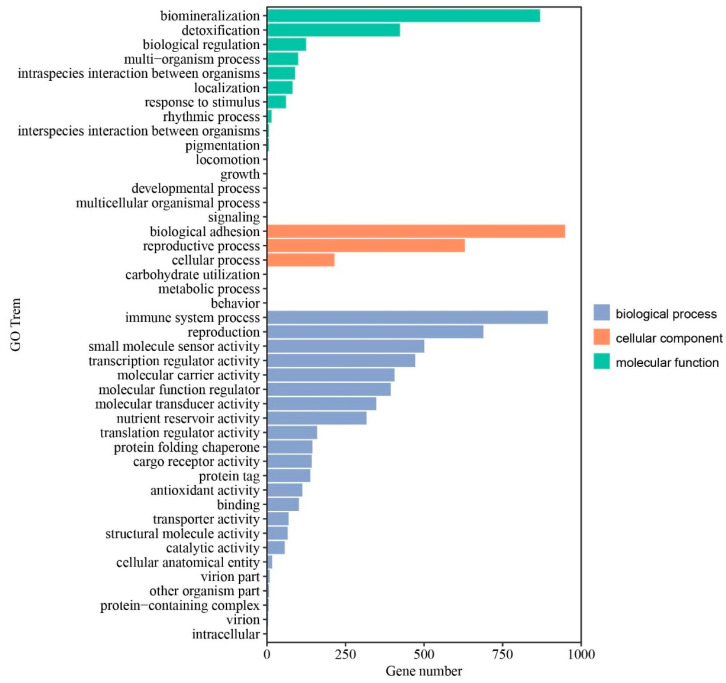
GO classification of ovarian DEGs. The longitudinal and horizontal coordinates represent the GO term and the number of DEGs annotated to the term, respectively. Green, orange and purple indicate the molecular function, cellular component and biological process, respectively.

Subsequently, KEGG and GO enrichment analyses were performed to determine the biological functions and key pathways of DEGs identified in ovaries. Functional classification of DEGs using KEGG pathway enrichment analysis demonstrated that the ovarian DEGs were associated with 209 pathways. The top 20 significantly enriched KEGG pathways are listed in Figure 3. We found that focal adhesion, vascular smooth muscle contraction, the MAPK signaling pathway, adrenergic signaling in cardiomyocytes, ECM–receptor interaction, the Notch signaling pathway, the apelin signaling pathway, the FoxO signaling pathway, the GnRH signaling pathway, and the Wnt signaling pathway in ovaries may play important roles in the broodiness process of Changshun hens. Notably, down-regulated vascular smooth muscle contraction, ECM–receptor interaction, and the GnRH signaling pathways were observed in incubating ovaries. GO enrichment analysis demonstrated that a total of 37 GO terms were enriched, including 12 molecular function terms, 3 cell components, and 22 biological process terms (Table S4). The top 10 significantly enriched GO terms were biomineralization, biological adhesion, immune system process, reproduction, the reproductive process, small molecule sensor activity, transcription regulator activity, detoxification, molecular carrier activity and the molecular function regulator (Figure 4). Taken together, our results indicated that these GO terms and KEGG pathways might play critical roles in the ovaries of Changshun hens during the transition from egg-laying to incubation.

### 3.4. Interaction Network Construction of DEGs

**Figure 5:**
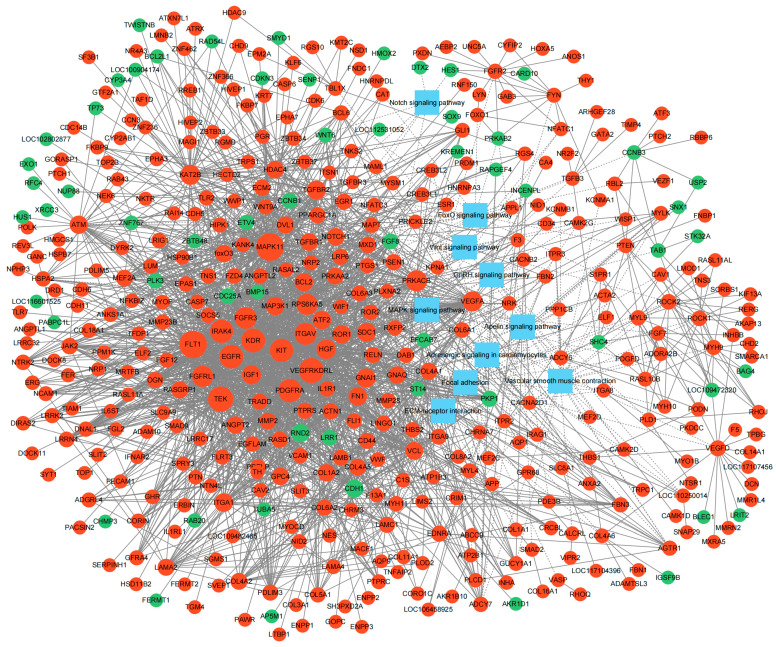
PPI networks of ovarian DEGs. Red and green circle nodes indicate down-regulated and up-regulated DEGs, respectively. The size of the node represents the expression of DEGs. Blue rectangles indicate the KEGG pathway. Interactions were shown as solid lines between proteins, and edges of KEGG pathway in dashed lines.

Ultimately, a PPI network of ovarian DEGs was constructed and visualized using Cytoscape to further identify hub genes associated with broodiness. As shown in Figure 5, the PPI network contained 455 nodes and 2392 edges, and were mainly enriched into 10 important pathways including focal adhesion, vascular smooth muscle contraction, the MAPK signaling pathway, adrenergic signaling in cardiomyocytes, ECM–receptor interaction, the Notch signaling pathway, the apelin signaling pathway, the FoxO signaling pathway, the GnRH signaling pathway, and the Wnt signaling pathway. The top eight hub genes with the highest interaction node degrees in the PPI network were *EGFR*, *VEGFRKDRL*, *FLT1*, *KDR*, *PDGFRA*, *TEK*, *KIT* and *FGFR3*, implying their potential roles in the transition from egg-laying to incubation in ovaries of Changshun hens.

### 3.5. Validation of DEGs by qRT-PCR

**Figure 6:**
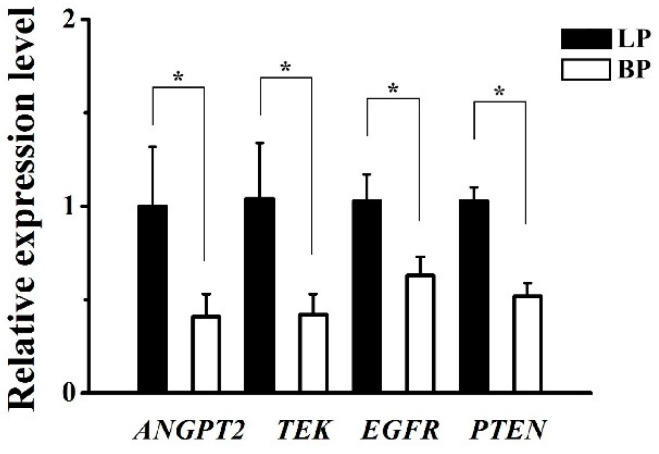
qRT-PCR validation of ovarian DEGs. The results were expressed as mean  ±  SD. * *p* < 0.05. LP, egg-laying group; BP, incubation group.

The results of the qRT-PCR showed that the expression levels of four mRNA selected were decreased significantly in the BP group compared with those in the LP group (Figure 6). It is inspiring that the expression trends validated via qRT-PCR were consistent with our RNA-Seq results, confirming that the RNA-seq results were reliable.

## 4. Discussion

Broodiness is known to be a maternal behaviour of poultry, and it is closely associated with HPGA. During the transition from egg-laying to incubation, the transcriptome changes of ovaries of Changshun green-shell laying hens, however, need to be further explored. In this study, we analyzed the ovarian transcriptome of Changshun hens at the LP and BP, and further identified the candidate genes and signal pathways related to the broodiness of Changshun hens through KEGG and GO enrichment analysis. This study not only provides novel insights into understanding avian broodiness, but also contributes valuable information to improve Changshun chicken breeding with low broodiness in the future.

Considering the fact that the avian ovary contains follicles at distinct developmental stages, including cortical follicles, white follicles, yellow follicles, and preovulatory follicles, it is, therefore, deemed to be an ideal model to study the mechanisms of follicular development [31,32]. Broodiness in avians is generally accompanied by the atresia of follicles and the degradation of reproductive performance. In the present study, we identified several critical signaling pathways related to follicle development in the ovaries of Changshun hens, including focal adhesion, the MAPK signaling pathway, the FOXO signaling pathway, and Wnt signaling. The transcriptome profile of geese revealed that ovarian development-related focal adhesion and ECM–receptor interaction were the top two pathways enriched with the most DEGs in incubating ovaries, indicating the potential central roles of these two pathways in the entire ovulation cycle [22]. Similarly, focal adhesion is found to play important roles in the prehierarchal follicles of laying and incubating geese [33]. In chickens, focal adhesion is observed to be closely associated with egg production and the process of follicle selection [34,35]. These findings indicate an important role of focal adhesion in regulating avian ovarian function and egg production. MAPK signaling pathway is one of the most important pathways in ovaries associated with avian broodiness and egg production [36,37,38,39]. The MAPK signaling pathway is demonstrated to be involved in cell proliferation and progesterone secretion of granulosa cells from the prehierarchical follicles in chickens [40,41,42]. Additionally, it is suggested that the granulosa layer within chicken follicles remains undifferentiated and steroidogenically inactive due to the inhibitory actions of MAPK and/or protein kinase C signaling [43]. The Wnt pathway is known to be an evolutionarily conserved signaling pathway. In humans, it has been shown to play a pivotal role during human follicle formation and follicle maintenance [44]. In domestic waterfowl, the Wnt signaling pathway might be implicated in the follicular development [17,33,45]. It was considered to be one of the most important signaling pathways in regulating broodiness of Muscovy ducks and Wanxi white geese [17,39]. In accordance with these findings, our results further indicated the important role of the Wnt signaling pathway in the ovaries of Changshun hens during broodiness. It was previously suggested that the FOXO signaling pathway in ovaries had important functions in the regulation of broodiness in ducks [15]. In the present study, we identified 20 DEGs, including PRKAB2, IGF1 and PTEN, classified into the FOXO signaling pathway. When incubation begins, chicken hens spend most of their time at the nest, and water and nutrient intake is drastically reduced, leading to a dramatic decrease in body weight. PRKAB2 is deemed to be associated significantly with feed conversion ratio, feed intake, and body weight [46]. It encoded the regulatory subunit of activated protein kinase (AMPK), which is a central regulatory factor in cellular energy metabolism [47]. Therefore, PRKAB2 as an AMPK subunit was involved in the fundamental regulation of the energy level within the cell and food intake at the whole body level [48]. Interestingly, a recent study demonstrated that AMPK regulated the activation of ovarian primordial follicles by modulating Wnt and FOXO, indicating a significant role in controlling the reproductive behaviour of animals [49]. IGF1 and PTEN have been reported to be potential key genes that regulate avian ovulation [50]. IGF-1 is expressed by granulosa and theca cells in avian ovary. It can stimulate avian granulosa and theca cell proliferation [51]. A variation of energy metabolism through AMPK activation was reported to modulate differently IGF-1-induced progesterone production in hen granulosa cells [52]. Therefore, IGF1, as an autocrine/paracrine regulator of follicular growth and differentiation, is involved in the regulation of avian follicular development [52,53,54]. PTEN is the phosphatase of phosphatidylinositol (3,4,5)-trisphosphate, which regulates cell proliferation cycles and inhibits cell migration. Broodiness is generally known to cause the atresia of follicles. Similarly, fasting can also induce the atresia of follicles, which is accompanied by a significant decrease expression of PTEN in chicken ovaries [55]. Further studies demonstrated that PTEN is involved in the ovarian function remodeling of laying hens via the KIT-PI3K-PTEN-AKT signaling pathway [55]. We found that both IGF1 and PTEN were down-regulated in incubating ovaries in the present study, indicating that they might have critical roles to play in the regulation of ovarian function in incubating chickens.

Vascular smooth muscle contraction, ECM–receptor interaction and the GnRH signaling pathways are classical signaling pathways associated with follicle development. Notably, vascular smooth muscle contraction, ECM–receptor interaction, and the GnRH signaling pathways in the present study were all down-regulated in the ovaries of incubating Changshun hens. The development of ovarian follicles generally requires the coordinated interactions between theca cells, oocytes and granulosa cells. Transcriptome analysis of circRNA and mRNA in theca cells from different types of follicles reveals the important roles of vascular smooth muscle contraction in follicular development in chickens [56]. In Taihe black-bone silky fowls, vascular smooth muscle contraction is deemed as a critical signaling pathway that would affect ovarian development at different egg-laying stages, and ECM–receptor interaction in ovaries might be essential for the transition from laying to broodiness [16,57]. In pigeons, ECM–receptor interaction and vascular smooth muscle contraction were reported to be closely related to promoting follicular maturation and ovulation in pre-ovulatory follicles [58]. In geese, vascular smooth muscle contraction was involved in follicular development from the F4 to F1 stage, and ECM–receptor interaction was significantly enriched in ovaries from the pre-laying period to the broody period [22,59]. The GnRH signaling pathway is known to be involved in the ovarian function of chickens [60]. In this study, a total of 14 down-regulated DEGs, including CACNA1C, GNAQ, ADCY5, and MMP2, were mapped to the GnRH signaling pathway. GNAQ and CACNA1C have been found to be associated with the development of ovarian follicles and the onset of the reproductive maturation in animals [61,62]. In chickens, the expression of GNAQ and CACNA1C were demonstrated to be regulated by MSTRG.19756.2, a novel lncRNA, in trans, and then they acted as upstream factors of the GnRH signaling pathway to affect the downstream genes involved in ovarian development [63]. ADCY5, as a member of the adenylatecyclases family, is reported to be responsible for egg production in Muscovy ducks [37]. Furthermore, ADCY5 has been identified to be closely associated with the ovarian morphological related traits of animals [64]. MMP2 is reported to be associated with ovary development in chickens [65]. It was reported that there was a lower expression of MMP2 in the atrophy of chicken ovaries [65].

It is now generally recognized that the control of avian ovarian development involves pituitary gonadotropins and various local paracrine and autocrine growth factors such as epidermal growth factor (EGF). In the present study, EGFR, the receptor of EGF, was observed to decrease significantly in ovaries from incubating Changshun hens. Similarly, a previous study revealed the dynamic expressions of EGFR in chicken ovarian follicles [66]. Transcriptomic analysis reveals that EGFR is abundantly but differentially expressed in granulosa cells proximal and distal to the germinal disc of chicken preovulatory follicles [67]. EGFR has been shown to be expressed highly in granulosa cells from the prehierarchical follicles, and thereafter, its expression is found to decrease markedly to the stage of the largest preovulatory follicles [66,68]. EGF functions as a ligand of EGFR to stimulate the proliferation of chicken granulosa cells [32,66,69]. However, the in vitro experiments have shown that EGF or FSH-induced proliferation of granulosa cells can be reversed by EGFR inhibitor AG1478 [66]. Similarly, AG1478 can also significantly inhibit EGF or FSH-reduced apoptosis of granulosa cells [66]. These results indicate the important roles of EGFR in chicken reproduction and broodiness.

The growth and maturation of ovarian follicles in chickens requires a complex network of blood vessels. Compared to prehierarchical follicles, which have a limited number and size of blood vessels, the large yellow follicles and preovulatory follicles are highly vascularized [53]. The vascular endothelial growth factor (VEGF), platelet-derived growth factor (PDGF), angiopoietin and their receptors are involved in angiogenesis. VEGF is a key regulator of physiological angiogenesis as it can facilitate blood vessel growth and remodeling processes. The network of ovarian blood vessels is closely associated with VEGF. VEGF exerts biological effects by binding to its tyrosine kinase family receptor FLT1, KDR, FLT4 and KDRL (also known as VEGFRKDRL) [70,71]. Interestingly, VEGF seemly binds to its receptors FLT1 and KDR with high affinity [72]. KDR is mainly expressed in the vascular endothelium of the theca layer in chicken follicles. KDR might be the most important receptor which is involved in VEGF-induced angiogenesis [73,74,75]. FLT1 can regulate VEGF activity via interacting with VEGF and making it less available to KDR [76]. Decreased expression of VEGF, FLT1 and KDR might be related to follicle atresia in chickens [53]. Consistently, results from the present study showed that there was a significant decrease in expression of VEGF, including VEGFA and VEGFD, and its receptors, FLT1, KDR and VEGFRKDRL, in ovaries from incubating Changshun hens, indicating the important roles of VEGF and its receptors in chicken broodiness.

Simultaneously, our results showed that there was a significant decrease in PDGF and its receptor (PDGFR) in the ovaries of incubating chickens compared with egg-laying hens. There are four PDGFs, including PDGFA, PDGFB, PDGFC and PDGFD, and two receptors, including PDGFRA and PDGFRB, identified in vertebrates [77,78]. The importance of PDGFRA has been confirmed by studies that showed the presence of PDGFRA in follicular cells in the ovaries of different species. In humans, PDGFRA is widely expressed in oocytes, theca cells and ovarian stroma cells [79]. In rats, PDGFRA is also identified in oocytes and granulosa cells [80]. The expression pattern of PDGFRA indicated that it might be involved in the ovarian folliculogenesis, selection and growth initiation of follicles and the formation of the thecal layer [81,82]. Meanwhile, PDGFRA is reported to be a requirement in steroid-producing cells in mice ovaries, and is involved in the steroidogenesis through regulating the downstream target genes Sgpl1, Plekha1, Tiparp, Schip1, and BC058969 [83]. Additionally, signaling downstream of PDGFRA has been reported to induce both apoptotic and antiapoptotic responses [84,85]. The identification of PDGFRA but not PDGFRB in ovaries from incubating Changshun hens in the present study further suggests that PDGFRA may be important for ovarian development during chicken broodiness.

TEK is a tyrosine kinase receptor and can bind with angiopoietins. Angiopoietin 1 (ANGPT1), angiopoietin 2 (ANGPT2), and the TEK receptor tyrosine kinase are mainly expressed in ovaries. In this study, we found that TEK and angiopoietins were down-regulated in ovaries from incubating Changshun chickens. The angiopoietin-TEK system is observed to play a crucial role in blood vessel formation and stability, follicular development and atresia [86,87]. The ANGPT1 and ANGPT2 can bind to TEK, inducing opposite effects. ANGPT1 elicits an activation of TEK by increased tyrosine phosphorylation of TEK when they bind. ANGPT2, as a natural antagonist, acts to inhibit the activation of TEK and disrupts ANGPT1-dependent TEK-mediated angiogenesis [88]. ANGPT2 is reported to destabilize existing vessels, loosening the supporting cell matrix to allow angiogenic factors such as VEGF to stimulate cell proliferation and migration during early angiogenesis [89]. Additionally, TEK is hypothesized to play an important role in folliculogenesis [88].

The functionality of the mammalian Kit system, composed of Kit ligand (KL) and its tyrosine kinase receptor (KIT), has been shown that they have multiple roles during oogenesis, folliculogenesis, and melanogenesis [90,91]. The ovarian expression pattern revealed that mammalian KIT is mainly expressed in theca cells, oocytes and follicular fluid [92,93,94]. Similarly, the chicken KIT is observed to express in very small follicles (<1 mm), theca cell layer and the ovarian stroma, indicating that the Kit system might promote the transition from quiescence to slow growing follicles in chickens [95]. Furthermore, KIT is reported to be detected in many other chicken organs, including the testis, brain, bursa, spleen, thymus, heart and kidney, indicating that the Kit system might be implicated in a variety of non-ovarian functions [96].

Fibroblast growth factor receptor 3 (FGFR3) is known to be a receptor for fibroblast growth factors (FGF). In mammals, FGFR3 is identified in oocytes, granulosa cells, theca cells and stromal cells [97,98]. Additionally, FGFR3 is expressed by human primordial germ cells during the first and second trimester, and is then repressed after meiotic initiation to form primordial oocytes [99]. A study on buffalos revealed that FGFR3 is expressed widely in ovarian follicles during different stages of development [100]. FGFs and their receptors are reported to be involved in multiple biologic processes in angiogenesis, hematopoiesis, wound healing and even embryonic development [101]. Studies based on transgenic mice models showed that mutant FGFR3 leads to dwarfism and infertility [102]. In chickens, FGFR3 is identified to be a candidate gonadal sex differentiation gene in embryos [103]. Decreased FGFR3 in incubating hens in the present study further supports the assumption that FGFR3 might play critical roles in chicken reproduction and broodiness.

The incubation rhythm of poultry is very flexible. It is not only influenced by endocrine and genetics, but also by various environmental stimuli. In domestic chickens, incubation behaviour is generally triggered by the accumulation of a sufficient number of eggs to incubate. When hens are removed from their nest and transferred to a different environment for several days, the expression of incubation behaviour can then be stopped [104]. The photoperiod is an important environmental factor affecting avian broodiness. Long photoperiods can usually improve avian egg-laying performance and decrease avian broodiness. Transcriptome analysis showed that the photoperiod could affect smooth muscle cell proliferation in ovaries, as well as extracellular matrix-related function in follicles [105]. Similarly, food deprivation and high temperature can also result in abnormal ovarian gene expression in poultry [106,107]. Therefore, it is crucial to reduce the adverse effects of physiological and environmental factors on the experimental results during the experimental process. In the present study, all animals were raised under equivalent conditions. The animals were provided with the same feeding regime for 24 weeks prior to ovary collection. Sample collection was performed by competent personnel who experienced and correctly applied the technique to minimize any distress or pain to animals.

In summary, the results based on the Changshun green-shell laying hen, a Chinese indigenous breed with strong broodiness, provide new insights into the ovarian regulation of incubation behaviour. Meanwhile, this study provides valuable information to improve Changshun hen breeding with low broodiness in the future. However, we have to admit that the study had limitations such as a small sample size, a single breed, and potential experimental biases. The analyses of the present study must be considered exploratory and will need to be further validated in bigger cohorts. Nevertheless, the results of the present study are still meaningful for exploring the mechanism of broodiness in poultry.

## 5. Conclusions

In this work, we characterized and evaluated the ovarian transcriptome in LP and BP Changshun hens. The results suggest that 1650 DEGs were identified in ovaries between laying and incubating Changshun green-shell laying hens, and eight key genes (*EGFR*, *VEGFRKDRL*, *FLT1*, *KDR*, *PDGFRA*, *TEK*, *KIT* and *FGFR3*) might take part in the broodiness of Changshun hens through focal adhesion, the MAPK signaling pathway, the FoxO signaling pathway, vascular smooth muscle contraction, ECM–receptor interaction and the GnRH signaling pathway in ovaries. Future work should elucidate how these key genes regulate avian incubation behaviour. The results from this study further confirm the complexity of the javascript:void(0);reproductive behaviour of different avian breeds. Meanwhile, our findings lay the groundwork for developing strategies to control broodiness in poultry, which could have significant implications for poultry production and breeding practices.

## Figures and Tables

**Table 1:** Data quality and mapping data statistics of RNA-seq libraries.

Sample	Clean Reads	Clean Bases	Q20 (%)	Q30 (%)	GC (%)	Total Mapped	Uniquely Mapped	Multiple Mapped
LP1	22,097,938	6,611,280,448	98.46	95.39	49.5	41,172,905 (93.16%)	40,260,429 (91.10%)	912,476 (2.06%)
LP2	19,277,102	5,768,363,046	98.54	95.51	48.36	36,607,544 (94.95%)	35,884,081 (93.07%)	723,463 (1.88%)
LP3	23,428,349	7,006,988,490	98.41	95.25	49.60	43,906,095 (93.70%)	42,884,740 (91.52%)	1,021,355 (2.18%)
BP1	20,509,236	6,134,357,836	98.45	95.56	51.22	38,159,435 (93.03%)	37,161,046 (90.60%)	998,389 (2.43%)
BP2	21,578,186	6,454,693,384	98.34	94.87	47.94	41,280,264 (95.65%)	40,498,880 (93.84%)	781,384 (1.81%)
BP3	20,424,383	6,112,551,828	98.46	95.21	47.89	38,963,103 (95.38%)	38,206,570 (93.53%)	756,533 (1.85%)

LP, ovarian samples of egg-laying group; BP, ovarian samples of incubation group; Q20, sequencing error rates lower than 1%; Q30, sequencing error rates lower than 0.1%; GC, the percentage of G and C bases in clean data.

## Data Availability

The raw sequence data in the present study have been deposited in the Genome Sequence Archive (Genomics, Proteomics and Bioinformatics 2021) in the National Genomics Data Center (Nucleic Acids Res 2022), the China National Center for Bioinformation/Beijing Institute of Genomics, and the Chinese Academy of Sciences (GSA: CRA016819) that are publicly accessible at https://ngdc.cncb.ac.cn/gsa (accessed on 6 June 2024).

## Supplementary Materials

The following supporting information can be downloaded at: https://www.mdpi.com/article/10.3390/genes16040394/s1, Table S1. Ingredients and nutrient composition of the experimental diets. Table S2. Primers used for qRT-PCR. Table S3. List of all the DEGs detected in ovaries. Table S4. List of GO enrichment analysis of DEGs.
