# Supplementary material for: Ovarian Transcriptome Profile from Egg-Laying Period to Incubation Period of Changshun Green-Shell Laying Hens
Source: Genes (Basel). 2025 Mar 29;16(4):394. doi: 10.3390/genes16040394 (PMC12026841; doi:10.3390/genes16040394)
Supplement: Supplementary file 1 [file genes-16-00394-s001.zip › Table S1. Ingredients and nutrient composition of the experimental diets.pdf]

Table S1. Ingredients and nutrient composition of the experimental diets.

**Ingredients (%)**	**Content**	**Nutrient level**	**Content**
Corn	64	Metabolic energy (mcal/kg)	2.96
Wheat shorts	2	Crude protein (%)	15.5
Rice bran	3	Lysine (%)	0.65
Bean pulp	9.7	Calcium (%)	1.19
Peanut bran	4	Phosphorus (%)	0.57
Corn gluten meal	2	Sodium (%)	0.206
Refined three worm powder CP45%	6	Chlorine (%)	0.196
Rock flour	1.5	Potassium (%)	0.47
Calcium hydrophosphate	1.3	Methionine (%)	0.25
Soybean oil	2.5	Arginine (%)	0.83
338 gunk	4		
Total	100		

Note: the vitamin/mineral premix includes (per kg feed): vitamin A, 15750 IU; vitamin D, 3500 IU; vitamin E 35 mg; Thiamine, 3.5 mg; Menadione, 4.4 mg; Riboflavin, 10.5 mg; vitamin B6, 7 mg; vitamin B12, 35 mg; Nicotinic acid, 70 mg; Folic acid, 1.75 mg; Pantothenic acid, 21 mg; Biotin, 0.175 mg.
