# Supplementary material for: Ovarian Transcriptome Profile from Egg-Laying Period to Incubation Period of Changshun Green-Shell Laying Hens
Source: Genes (Basel). 2025 Mar 29;16(4):394. doi: 10.3390/genes16040394 (PMC12026841; doi:10.3390/genes16040394)
Supplement: Supplementary file 1 [file genes-16-00394-s001.zip › Table S2. Primers used for qRT-PCR.pdf]

Table S2. Primers used for qRT-PCR.

Gene Name	Primer Sequence (5'-3')
ANGPT2	ATGCTTGTGGTCCTTCTA<br>TCCTGAGCCTTTCCAGTA
TEK	GTTCTGCCTACCAGATCCA<br>TGCAGTCTGACCCATAATA
EGFR	CTGGTTGTGGTTGG<br>GCGCTTCCGCACGATATG
PTEN	CCAGTCAGAGACGCTAC<br>ATACCGGCAAAGGTTGA
β-actin	GAGAAATTGTGCGTGACA<br>CCTGAACCTCTCATTGCCA
