# Supplementary material for: Ovarian Transcriptome Profile from Egg-Laying Period to Incubation Period of Changshun Green-Shell Laying Hens
Source: Genes (Basel). 2025 Mar 29;16(4):394. doi: 10.3390/genes16040394 (PMC12026841; doi:10.3390/genes16040394)
Supplement: Supplementary file 1 [file genes-16-00394-s001.zip › Table S4. List of GO enrichment analysis of DEGs.pdf]

Table S4. List of GO enrichment analysis of DEGs

GO category	GO subcategory	Number of DEGs
Cellular component	Intracellular	630
	Protein-containing complex	215
	Cellular anatomical entity	949
Molecular function	Catalytic activity	424
	Structural molecule activity	61
	Transporter activity	82
	Binding	870
	Antioxidant activity	7
	Cargo receptor activity	15
	Translation regulator activity	7
	Nutrient reservoir activity	1
	Molecular transducer activity	90
	Molecular function regulator	100
	Transcription regulator activity	125
	Small molecule sensor activity	1
Biological process	Reproduction	145
	Immune system process	113
	Behavior	69
	Metabolic process	501
	Cellular process	894
	Reproductive process	143
	Biological adhesion	102
	Signaling	348
	Multicellular organismal process	406
	Developmental process	394
	Growth	57
	Locomotion	160
	Pigmentation	6
	Interspecies interaction between organisms	66
	Rhythmic process	17
	Response to stimulus	472
	Localization	317
	Intraspecies interaction between organisms	4
	Multi-organism process	138
	Biological regulation	689
	Detoxification	7
	Biomineralization	9
